# *EWSR1::ATF1* Translocation: A Common Tumor Driver of Distinct Human Neoplasms

**DOI:** 10.3390/ijms252413693

**Published:** 2024-12-21

**Authors:** Julia Raffaella Bianco, YiJing Li, Agota Petranyi, Zsolt Fabian

**Affiliations:** 1 School of Medicine and Dentistry, Faculty of Clinical and Biomedical Sciences, University of Central Lancashire, Preston PR1 2HE, UK; jrbianco@uclan.ac.uk (J.R.B.); yjli4@uclan.ac.uk (Y.L.); 2 Centre of Excellence for Pancreatic Diseases, Semmelweis University, 1083 Budapest, Hungary; petranyi.agota@semmelweis.hu; 3 Translocon Biotechnologies PLC, Akademia u. 6, 1056 Budapest, Hungary

**Keywords:** Ewing sarcoma region 1, activating transcription factor 1, reciprocal chromosome translocation, gene fusion, chimeric proteins, malignant mesothelioma

## Abstract

Cancer is among the leading causes of mortality in developed countries due to limited available therapeutic modalities and high rate of morbidity. Although malignancies might show individual genetic landscapes, recurring aberrations in the neoplastic genome have been identified in the wide range of transformed cells. These include translocations of frequently affected loci of the human genetic material like the Ewing sarcoma breakpoint region 1 (*EWSR1*) of chromosome 22 that results in malignancies with mesodermal origin. These cytogenetic defects frequently result in the genesis of fusion genes involving *EWSR1* and a number of genes from partner loci. One of these chromosomal rearrangements is the reciprocal translocation between the q13 and q12 loci of chromosome 12 and 22, respectively, that is believed to initiate cancer formation by the genesis of a novel, chimeric transcription factor provoking dysregulated gene expression. Since soft-tissue neoplasms carrying t(12;22)(q13;q12) have very poor prognosis and clinical modalities specifically targeting t(12;22)(q13;q12)-harboring cells are not available to date, understanding this DNA aberration is not only timely but urgent. Here, we review our current knowledge of human malignancies carrying the specific subset of *EWSR1* rearrangements that leads to the expression of the EWSR1::ATF1 tumor-driver chimeric protein.

## 1. Introduction

Approximately 19.3 million new cases and 10 million cancer-related deaths were estimated in 2020 [1]. Cancer is a multistep disease that begins with DNA damage, which is a key driver of tumorigenesis. The most critical types of DNA damage are DNA double-strand breaks (DSBs) that can arise from both exogenous and endogenous impacts like ionizing radiation and DNA replication errors, respectively. Moreover, DSBs can be part of programmed cellular processes like the V(D)J recombination or meiotic exchange of homologous loci. Unrepaired single-strand breaks (SSBs) can also escalate into one-ended DSBs, ultimately leading to the collapse of replication forks [2]. SSBs are closely associated with oxidative stress and may occur indirectly during the repair of oxidized bases or directly via the oxidative fragmentation of the phosphate–deoxyribose backbone [3]. Unrepaired DSBs typically result in cell cycle arrest and apoptosis, while faulty repair can lead to carcinogenesis by initiating structural chromosomal aberrations including inversions, deletions or translocations.

In eukaryotic cells, DSBs are primarily repaired via two mechanisms: the non-homologous end joining repair, which repairs damaged ends with minimal sequence homology, and homologous recombination, which utilizes extensive homology to restore the original sequence [4]. Erroneous repair of homologous loci between unrelated chromosomes is believed to be one of the potential mechanisms of reciprocal chromosome translocations [5].

Chromosome 22, the smallest chromosome containing only 52 Mb of DNA and the first one that was completely sequenced, is frequently involved in structural rearrangements with pathological consequences [6]. The vast amount of low copy repeats found in chromosome 22 have been linked to its high number of pathologies [7]. Indeed, 10% of the well-conserved chromosome 22 consists of segmental duplications [7,8]. In total, 19% of the genes of chromosome 22 are pseudogenes that, while showing a high degree of similarity to known genes, usually do not produce proteins due to their interrupted reading frames [9]. In addition, 47.2% of chromosome 22 contains CpG islands, which is significantly higher than the 42% found across the human genome, although fluctuations in this amount have been noted [9]. These characteristics are believed to be the major determinants for chromosome 22 being a hot spot for chromosome translocations.

One of the most well-known outcomes of chromosome 22 rearrangements is the genesis of Philadelphia chromosome in which the reciprocal translocation between chromosome 22 and chromosome 9 results in the formation of the *BCR::ABL* fusion gene, a hallmark of chronic myeloid leukemia (CML) [10,11,12]. The q22.2 region of chromosome 22 harbors another mutational hot spot known as the Ewing sarcoma breakpoint region 1 (*EWSR1*) that is involved in reciprocal translocations with a number of chromosomes. These include chromosome 11, resulting in the *EWSR1::FLI1* chimera, which is pathognomonic for Ewing sarcoma, as well as q13 segments of chromosome 12 that, when fused to the q12.2 locus of chromosome 22, result in the genesis of another chimeric gene, the *EWSR1::ATF1* [13,14,15].

The t(12, 22)(q13;q12) rearrangement is most commonly associated with the clear cell sarcoma (CCS) of adolescent people but it has also been detected in other pediatric malignancies including malignant mesothelioma, intracranial non-myxoid angiomatoid fibrous histiocytoma (AFH) and atypical central neurocytoma [16,17,18,19]. In addition, several adult tumors have also been linked to the t(12, 22)(q13;q12) rearrangement including testicular sex cord tumors, primary pulmonary myxoid sarcoma and intracranial myxoid mesenchymal tumors just to name a few [20,21,22]. Here, we review our current knowledge of the t(12, 22)(q13;q12) rearrangement and its clinical consequences.

## 2. The *EWSR1* Gene

**Figure 1:**
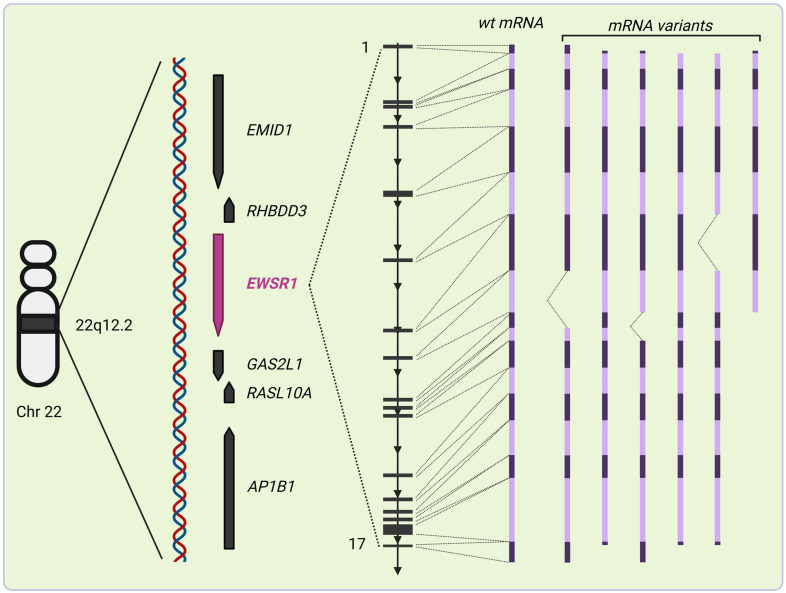
Structure of *EWSR1*. *EWSR1* spans about 40 kb within the 12.2 locus of chromosome 22. It is most closely surrounded by genes in both forward and reverse orientations encoding nuclear proteins involved in interactions between chromosomes and the cytoskeleton (*GAS2L1*), and inhibition of cellular proliferation (*RASL10A*), as well as *RHBDD3* that encodes an integral membrane protein predicted to be involved in protein metabolism. Its 17 exons generate a primary transcript that can give rise to various mature mRNAs by alternative splicing. Many of them, apparently, dictate translation of the corresponding polypeptides. The most well-documented alternative transcripts are depicted in the figure. Different colors of transcript variants represent spliced neighboring exons. The figure was created with Biorender.com.

The *EWSR1* gene is located on the q12.2 locus of chromosome 22 (Figure 1) [6]. The wild-type *EWSR1* encodes a 656 amino acid protein that belongs to the highly conserved FET family of proteins, which includes the Fused in Sarcoma/Translocated in Liposarcoma (FUS) protein and the TATA-box binding protein Associated Factor 15 (TAF15) [23]. Although *EWSR1* is an evolutionary well-conserved gene, it has a sizeable quantity of heterogeneity at the 5′ end [24]. Its promoter region usually lacks a TATA box and has a high incidence of unmethylated CpG nucleotides supporting the idea of the existence of multiple transcription start sites (TSS) [24]. These characteristics are believed to play a role in the low tissue specificity of the *EWSR1* transcription [25].

Previous research has demonstrated that WNT inhibitors can repress the expression of genes involved in epithelial–mesenchymal transition genes including *EWSR1,* suggesting that WNT/β-catenin signaling is one of the regulatory pathways of *EWSR1* [26,27,28]. WNT signaling has also been linked to the regulation of the expression of several transcription factors including *AP1*, *AP2*, *CMYB* and *PEA3* [29,30,31,32,33]. Interestingly, investigation of *EWSR1* in the TFDSITE database also identified binding sites of AP2, SP1, C-MYB and PEA3 in the first exon of *EWSR1* but it is unclear which of these transcription factors, if any, are genuinely involved in the regulation of the transcription of *EWSR1* [24].

The *EWSR1* transcript spans 40 kb and consists of 17 exons and 16 interrupting introns (Figure 1) [24]. Intron 6 is a region identified as an active area of retro-transposition and recombination [34]. Intron 6 lays directly upstream to the *EWSR1* region and may be associated with frequent somatic recombination of this locus [34]. Further downstream of exon 6, the *EWSR1* region is flanked by 7 kb of DNA of exon-8, -9 and -10 that are regularly disrupted in Ewing sarcoma with common breakpoints in intron-7 and -8 [24,25].

Alternative splicing is seen in the *EWSR1* transcript (Figure 1) [35]. So far, 22 alternatively spliced transcripts of *EWSR1* have been described; 7 of them do not produce proteins [36]. The alternative splicing of *EWSR1* may play a role in the pathogenesis of associated tumors and transcript variants might be differentially expressed in various tumor types, influencing tumor behavior and progression [35].

Although little direct experimental data are available on the characteristics of the translation of *EWSR1* transcripts, one of the alternative transcripts directs the translation of a protein named EWSR1-b, which appears to have similar functions to EWSR1 but with increased affinity to RNA at the poly G and poly U regions [35].

Post-translational modifications of the de novo translated EWSR1 have also been observed. Indeed, ERK1/2, as well as other threonine protein kinases, has been linked to the control of the expression of *EWSR1* [37]. Increased phosphorylation of EWSR1 by threonine protein kinases induces overexpression of the protein during exposure to DNA-alkylating agents [37]. Since threonine phosphorylation is also present in chimeric EWSR1 proteins, one can speculate if a similar responsiveness of mutant EWSR1 to DNA damage exists [37].

Although members of the FET protein family might serve as an analogy of EWSR1 translation and post-transcriptional modifications, EWSR1 is the one least prone to cytoplasmic accumulation, suggesting significant physico-chemical, and, thus, functional, differences between FET family members [38]. Indeed, FUS and EWSR1 share very few gene targets, which perpetuates the narrative that FET proteins, although seemingly having similar functions in principle, are not redundant [39].

The full-length EWSR1 is enriched in alanine, threonine and proline while depleted in glycine compared to that of the other two members of the FET family [40]. In addition, although all three members contain a low complexity domain (LCD) at their N-termini, its amino acid composition and behavior in EWSR1 differs from that of the FUS and TAF15. In EWSR1, distribution of alanine and serine is predominantly clustered in the N- and C-terminus of the LCD [40,41]. Tyrosine residues of this region, which also show characteristic distribution, play a pivotal role in the formation of hydrophobic intra- and intermolecular interactions [40]. Experimental data indicate that these molecular signatures result in a dynamic and disordered N-terminus that lacks secondary structure but is more prone to phase separation, facilitating the formation of droplets with liquid-like nature [40]. The observation that the EWSR1 LCD-mediated droplet formation occurs under the physiologic pH range suggests a functional role of the LCD domain of EWSR1 [40].

**Figure 2:**
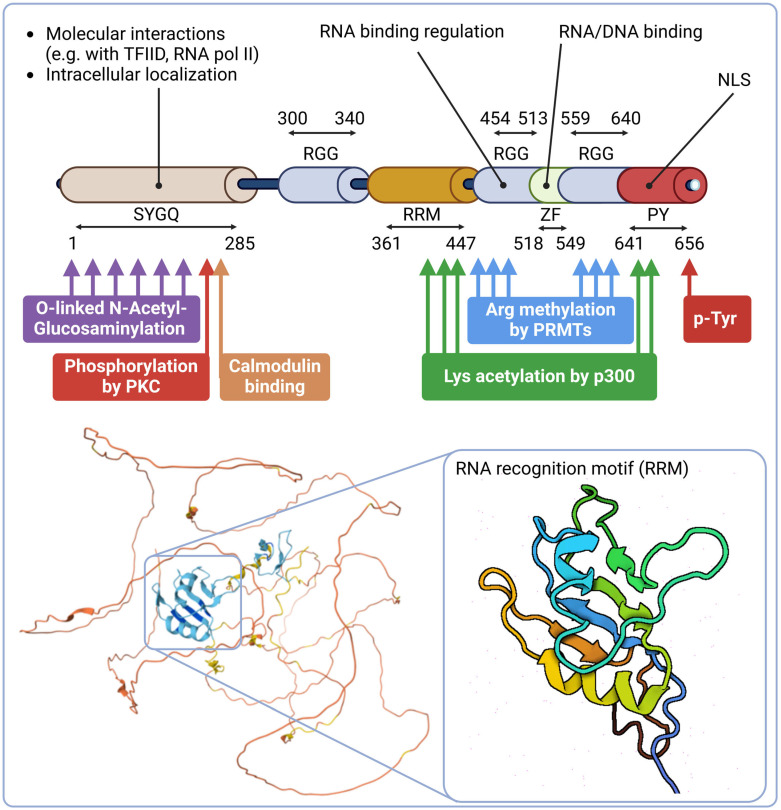
Structure and functions of EWSR1. Wild-type EWSR1 has an N-terminal low complexity domain (LCD) that is mainly composed of serine–tyrosine–glycine–glutamine (SYGQ) repeats. The LCD is the subject of extensive post-translational glycosylations and phosphorylations. The C-terminal half consists of multiple domains that affect EWSR1 affinity to distinct nucleic acid species. These include three arginine–glycine–glycine-rich domains (RGG) flanking a conserved RNA recognition motif (RRM) and a zinc finger domain (ZF). The RRM consists of four anti-parallel β-strands and two α-helices arranged in a β-α-β-β-α-β fold with side chains that stack with RNA bases. Specificity of RNA binding is determined by multiple contacts with surrounding amino acids in the RGG and ZF domains [47]. These interactions are affected by multiple post-translation modifications of the RGG and ZF motifs including arginine methylations and lysine acetylations, respectively. The figure was created with Biorender.com.

Indeed, EWSR1 LCD has been shown to be required for transformation in Ewing sarcoma models, suggesting that the LCD-mediated self-association and phase separation is an important step in EWSR1-mediated oncogenesis [42,43,44]. Since experimental data indicate that EWSR1 plays a role in transcription, DNA repair, cell division and cellular senescence, the multivalent nature of the EWSR1 LCD seems to be an important mediator of the underlying putative protein–protein and protein–RNA interactions [23,45,46]. In accordance, experimental data indicate that the N-terminus, encoded by exons 1-7, mediates transcriptional activation through degenerate repeats of the SYGQP motif via interaction with the basal transcription factor TFIID and RNA Polymerase II alike (Figure 2) [29].

Another difference between EWSR1 and its fellow protein family members is the dynamic and reversable O-linked β-*N*-acetylglucosamine (*O*-GlcNAc) modification of serine and threonine residues within the EWSR1 LCD [38,48]. Interestingly, 27 of the 37 tyrosines in the EWSR1 LCD are located immediately adjacent to potential O-linked N-acetylglucosaminylation sites [40]. Since, in other proteins, *O*-GlcNAc modification is usually linked to altered hydrophilicity, one can speculate that the addition of a sugar moiety to the LCD within the neighborhood of tyrosine residues reduces the probability of tyrosine-mediated hydrophobic molecular interactions, thus tuning the phase separation propensity [38,40]. To support this concept, the O-linked β-*N*-acetylglucosamine glycosylation of EWSR1 has been found to affect EWSR1 intracellular localization. During adipogenic differentiation, for instance, EWSR1 is, dominantly, localized to the nucleus. In adipocyte precursors, however, O-linked β-*N*-acetylglucosamine glycosylation activity is repressed and that is accompanied by the cytoplasmic abundance of EWSR1 [49]. A similar trend has been observed during neuronal differentiation, where the glycosylated state of EWSR1 correlates with the differentiation process and is mainly present in the early stages of development [50]. The importance of the nuclear localization of EWSR1 is further underlined by the observation that, in *EWSR1* knockout mice, the size of the nuclei is reduced in the neurons of the motor cortex, the striatum and the hippocampus, suggesting tissue-specific roles of EWSR1 in the central nervous system [51]. Indeed, experimental data indicate that EWSR1 is involved in the control of the alternative splicing of *FOXP1*, a master transcriptional regulator during neuronal differentiation, so one can speculate that EWSR1, at least in part, mediates tissue-specific RNA biogenesis [52].

Further analyses of EWSR1 strongly support this concept as, in its mid-region, EWSR1 harbors an 87 amino acid-long RNA recognition motif (RRM) encoded by exon-11, -12 and -13, a zinc finger region that binds both RNA and DNA and multiple Arg-Gly-Gly (RGG)-rich spans that modulate RNA binding (Figure 2) [53]. Data indicate that both the RRM and RGGs are required for the molecular interactions they catalyze [53]. Indeed, this region of EWSR1 seems to form a flexible RNA-binding domain (RBD) that employs a concave surface on the RRM to bind DNA G4s and RNA:DNA hybrids and residues of RGGs synergize with the RRM to increase EWSR1 affinity to hybrid nucleic acid structures [53]. In accordance, EWSR1 has been found to bind thousands of mRNAs to exert its regulatory activities in RNA metabolism via multiple mechanisms [54]. Binding EWSR1 to the 3′ untranslated region (3′ UTR) of some mRNAs, like the ones encoded for Cofilin-1 or PRAS40, represses their nuclear export and consequent cytoplasmic translocation [55,56]. EWSR1 has also been found to interact with the 5′ untranslated regions (5′ UTR) of transcripts, like *DROSHA*, where EWSR1 binding represses *DROSHA* transcription, suggesting a repressor role of EWSR1 in miRNA processing [57,58]. In accordance, miR-29b and miR18b have been found to be upregulated in *EWSR1*^−/−^ cells, leading to the consequent downregulation of critical mediators of dermal development *COL3A1* and *CTGF* [58]. *EWSR1*^−/−^ cells also show the accumulation of miRNA *let-7g* precursors, resulting in the downregulation of its mature form, a hallmark of Ewing sarcoma cells, underlining the role of EWSR1-mediated miRNA biogenesis in the context of oncogenesis [59]. In return, EWSR1 expression is repressed post-transcriptionally by the upregulation of miR-141 and miR-200b during neuronal differentiation, indicating the existence of multiple negative feedback loops in the EWSR1-mediated miRNA metabolism [60].

RGG boxes of its RBD can also repress the transcriptional activity of EWSR1 [61]. Their arginine residues can undergo methylation by type 1 protein arginine methyltransferases (PRMTs) in vivo [62]. RGG methylation results in the exclusion of EWSR1 from the nucleus that not only renders it cytoplasmic but can also facilitate its translocation to the cell surface [63,64]. These measures, however, seem to be cell-type-specific, since EWSR1 appears abundant in the cytoplasm only in certain cell types like secretory cells [65]. Lysines within 423–643 residues can undergo acetylation, mainly, by p300 that affect the splicing activity of EWSR1 in an, apparently, cell cycle-dependent manner [66]. It has been shown that this modification leads to the skipping of exon-1 of checkpoint kinase 2 (*CHEK2)* transcripts, preventing *CHEK2* translation upon UV-mediated DNA damage, indicating a role of EWSR1-mediated RNA metabolism in the DNA damage response as well.

Between its amino acid residues of 258 and 280, EWSR1 contains an IQ (isoleucine–glutamine) domain, a conserved motif found in a variety of proteins that are involved in calcium signaling [67]. The characteristic sequence pattern within the domain typically consists of around 25 amino acids and is known for its ability to bind calmodulin [68]. EWSR1 interaction with calmodulin suggests that EWSR1 is connected to the calcium-mediated signaling pathways. In accordance, the IQ domain of EWSR1 is identified as the specific site for PKC-mediated phosphorylation on serine 266 [67,69]. PKC-mediated phosphorylation of EWSR1 blocks its binding to RNA homopolymers and, inversely, formation of the EWSR1-RNA complex prevents EWSR1 phosphorylation by PKC [67]. The PKC-phosphorylated IQ domain of EWSR1 is frequently absent from chimeric mutants, raising the question of if the loss of PKC-mediated regulatory mechanism is involved in EWSR1-mediated oncogenesis [67].

In addition to RNAs, EWSR1 can bind to unusual DNA structures as well like the promoter of *CSFLR* that lacks the TATA box, CCAAT box and GC-rich spans alike [70]. *CSFLR* encodes for the macrophage growth factor CSF-1 and the observation that *EWSR1*-null mice exhibit severe and progressive atrophy of the hematopoietic organs, as well as decreased lymphoid and increased myeloid cell pools, indicates the complex and general roles of EWSR1 in the terminal differentiation of the hematopoietic cellular elements, at least in part, via the regulation of gene expression [65,71].

Data from cell-free systems also indicate that EWSR1, similarly to other FET family members, can catalyze homologous DNA pairing, influencing DNA recombination [72]. This finding is in accordance with the observations that *EWSR1*^−/−^ spermatocytes are not able to form XY bivalents, thus failing meiotic recombination [73].

The C-terminus of EWSR1 is flanked by a proline–tyrosine nuclear localization signal (NLS) (Figure 2) [45,74]. In part, phosphorylation of tyrosine residues at position 656 within the NLS seems to be another important determinant for transportin-1-mediated nuclear localization of EWSR1 as, in its absence, EWSR1 accumulates in the cytosol [75].

## 3. The t(12;22)(q13;q12) Translocation

Chromosome rearrangements involving the *EWSR1* locus are believed to be the most frequent ones in clinicopathologically diverse soft-tissue tumors, including certain carcinomas and mesotheliomas [76]. In pathologic conditions, the majority of the *EWSR1* breakpoints fall in intron-6, -7 or -8, resulting in the in-frame fusion of the EWS N-terminus to, most commonly, DNA-binding domains of its fusion partners, resulting in chimeric proteins [77,78]. Intron-6 contains 11 Alu elements due to expansion via subsequent retro-transpositions until approximately 5 million years ago [34]. Then, likely due to a homologous recombination event, 2480 bases were deleted from intron-6 of *EWSR1,* giving rise to a smaller allele with only four Alu elements remaining [34]. This smaller allele is more frequent in populations with African origin where Ewing sarcoma incidence is tenfold lower than in Caucasian populations where the small *EWSR1* allele is not present, suggesting the critical role of intronic Alu elements in *EWSR1*-related chromosome rearrangements [34].

Common fusion partners of *EWSR1* are loci-encoding members of the cyclic AMP response element-binding (CREB) protein family. Upon these rearrangements, the N-terminal domain of EWSR1 is fused to the bZIP domains of CREB family members CREB1, CREM or ATF1 [79,80,81,82].

## 4. The *ATF1*

**Figure 3:**
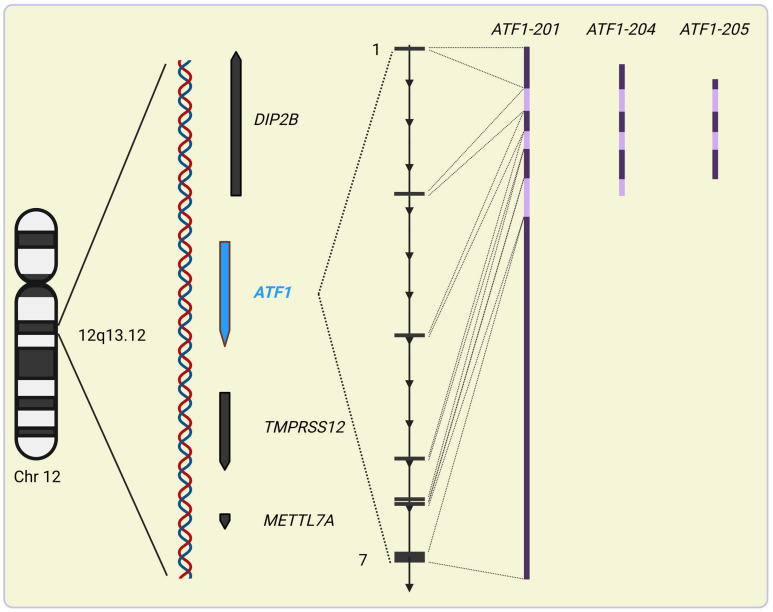
Structure of *ATF1*. *ATF1* spans about 57 kb along the plus strand of the q13.12 locus of chromosome 12. It is most closely surrounded by genes in similar forward orientations encoding a transmembrane serine protease (*TMPRSS12*) involved in the regulation of chromosomal synapsis formation and double-strand break repair, and *DIP2B* encoding a polypeptide that is predicted to participate in DNA methylation, up- and downstream, respectively. The seven exons of *ATF1* generate a primary transcript that, via alternative splicing, can give rise to three protein-coding mature mRNAs (*ATF1-201*, *-204* and *205*) and a minimum of two additional transcripts (*ATF1-202* and -*203*) that undergo nonsense mRNA-mediated decay. Different colors of transcript variants represent spliced neighboring exons. The figure was created with Biorender.com.

*ATF1* encodes the 271 amino acid of the bZip family member cyclic AMP (cAMP)-dependent activating transcription factor 1 (ATF1) [83]. The 57 kb long gene is located on chromosome 12 within the 12q13 locus flanked by the *DIP2B* and *TMPRSS12* genes (Figure 3) [72,84]. The wild-type *ATF1* has numerous intronic repeat sequences including both long and short interspersed nuclear elements (LINEs and SINEs, respectively) of which the latter ones have multiple Alu family members [84]. cDNA analysis of *ATF1* revealed that the transcript is composed of seven exons divided by six introns of which acceptor sites match the canonical GT/AG sequence [85]. Exon-2 and -7 are the shortest (99 bp) and longest (280 bp), respectively. Also, it is exon-2 that harbors the initiation *ATG* [86]. *ATF1* can give rise to three protein-encoding transcripts (*ATF1-201*, *ATF1-204 and ATF1-205*) while two other transcripts undergo nonsense mRNA-mediated decay (Figure 3) [77].

Translation of the *ATF1* mRNA is, at least in part, regulated via interactions between its 3′ UTR and miRNAs. The 3′ UTR of the *ATF1* mRNA harbors a binding site for microRNA-34c, a highly conserved miRNA that is expressed in the mature testis of both primates and rodents [87]. Although binding of microRNA-34c results in marked repression of the *ATF1* promoter activity in cell-free reporter assays, its silencing has been observed to upregulate ATF1 protein levels, leaving *ATF1* mRNA levels unaffected, suggesting that the microRNA-34c-mediated regulation of *ATF1* is rather post-transcriptional in vivo [87].

The ubiquitously expressed 271 amino acid-long ATF1 possesses C-terminal basic and leucine zipper motifs that together form a domain (bZIP) that shows a high degree of sequence homology with CREB [88]. bZIP, encoded by exon-6 and -7, mediates direct interaction with DNA and enables ATF1 to form homo- or heterodimers with other bZIP-containing proteins, including C/EBP, AP-1 or members of the MAF family [88,89]. The bZIP domain of ATF1 homo- or heterodimers binds the consensus sequence of GTGACGT(A/C)(A/G) of cAMP-inducible promoters [86,90,91,92]. DNA binding of wild-type ATF1 is under the control of a number of interacting partners including BRCA1 or calmodulin kinase I, and IV influencing ATF1 transcriptional activity [93,94,95].

Upstream of bZIP, between the 184 and 209 threonine residues, proteomics analyses proposed a number of putative phosphorylation sites. The proline-directed phosphorylation of threonine 184 by peptidyl-prolyl isomerase PIN1 was shown to post-transcriptionally stabilize ATF1 expression [96]. Elevated levels of Thr184 phosphorylation accompanied by increased activity of Matrix Metalloproteinase 2 (MMP) in gastric cancer metastases suggest the relevance of this phosphorylation in ATF1-mediated oncogenesis in vivo [97].

Unlike its C-terminus, the N-terminal region of ATF1 is fundamentally different to that of CREB, ATF1’s closest homolog. The N-terminal region (NTR) of ATF1 lacks both the glutamine-rich domain and the alpha peptide region of CREB, which are critical for the potent cAMP-induced transcriptional activity of the latter one [98,99]. In contrast, ATF1 NTR, apparently, can accommodate phosphorylation at serine 4, 5 and 6 that reduces the stability of the DNA binding of ATF1 [91,100]. Downstream of its NTR, ATF1 has a phosphorylated kinase-inducible domain (pKID) encoded by exon-3 that comprises the phosphoacceptor site for protein kinase A (PKA) flanked by two distinct elements called PDE1 and PDE2 upstream and downstream of PKA, respectively [86,91]. pKID is extensively phosphorylated by various kinases including Casein kinase I and II, CDK3 and MEK1, influencing the transcriptional activity of ATF1 [91,101,102,103]. Despite their high sequence homology in most coding regions, including phosphorylation sites for calmodulin-dependent kinases I and II, at serine 83 of ATF1 and serine 133 of CREB, unique N-terminal domains likely contribute to their specific transcriptional activities in response to different signals, thereby facilitating selective gene activation [104]. Indeed, ATF1 demonstrates relatively modest cAMP-induced activity compared to CREB [104].

ATF1 plays a critical role in regulating genes involved in cell proliferation, differentiation and survival [89,105]. Mapping ATF1 binding sites and target genes identified over 15,000 ATF1 binding sites and more than 350 genes involved in intricate feedback loops [106].

The *BRCA1* gene encodes proteins that are crucial in maintaining genome integrity and transcription regulation. A key feature of BRCA1 comprises a highly conserved RING domain, spanning the first 64 amino acids, which facilitates protein–protein interactions. BRCA1 behaves as a coactivator, interacting with ATF1 via its N-terminal 101 residues with the ATF1’s basic zipper DNA-binding domain. This interaction is further stabilized by coactivators CREB-binding protein and p300 [93]. BRCA1 enhances ATF1’s role in the DNA damage response and the transcription of target genes, such as H-2Dd, a major histocompatibility complex class I gene that facilitates the immune tagging of breast cancer cells for destruction [107]. This role likely extends to cooperation with CREB and ATF1 proteins in modulating TNF-α production during both hypoxia and radiation damage [93]. Missense mutations, such as the common C61G variant, disrupt the function of BRCA1 protein and contribute to treatment resistance, including Olaparib and cisplatin [93,108]. Although mutated, the C61G variant retains the ability to interact with and activate ATF1, thereby enhancing treatment resistance through promoting the transcription of genes involved in cellular proliferation and survival, such as those in the MAPK, NFκB and Wnt pathways [108].

Overexpression of ATF1 is also associated with the upregulation and expression of matrix metalloproteinase 2, contributing to metastatic phenotype in both nasopharyngeal carcinoma and melanoma cells [109].

Among others, ATF1 regulates the expression of *BCL2* and *BAX* and knockdown of ATF1 decreases the BCL2/BAX ratio in spermatocytes [87]. Interestingly, in vitro studies have also shown that the ATF1-regulator microRNA-34c acts as a pro-apoptotic factor that induces and represses *BAX* and *BCL2*, respectively, suggesting an ATF1-mediated counter measure of the apoptotic function of microRNA-34c [87]. ATF1 also participates in immune responses by regulating T cell proliferation and cytokine production [105]. A number of T cell-specific genes, like the ones encoding for T cell receptor α, interleukin-2 and CD8α, contain CRE elements in the promoter regions, suggesting an important role for CRE-binding transcription factors in T cell functions. The activation of CREB family transcription factors, including ATF1, is required for T cell proliferation and the regulation of cytokine production, such as IFN-γ, as observed in mouse models expressing a dominant negative form of CREB1 under a T cell-specific promoter [105,110].

The HRA and HRR regions of ATF1 serve to activate and suppress meiotic recombination, respectively [111]. The HRA region spans residues 151–225 and is necessary for promoting recombination activity [111]. Deletions of the left or right half of this region diminish its functionality by approximately half, suggesting distinct functional elements within this region. Three conserved sequence motifs, HRA-1, HRA-2 and HRA-3, work synergistically to activate recombination [111]. In contrast, the HRR region located between residues 251 and 325 of ATF1 is essential for repressing recombination [111]. One conserved sequence motif, HRR-1, is embedded in the HRR region [111]. The left or right ends alone are inadequate for achieving repression, suggesting that repressive activity requires the integrity of the entire sequence, particularly the central region [111]. Both HRA and HRR function independently and can act antagonistically to regulate the recombination activity of ATF1 [111].

## 5. The EWSR1::ATF1 Fusion Gene

**Figure 4:**
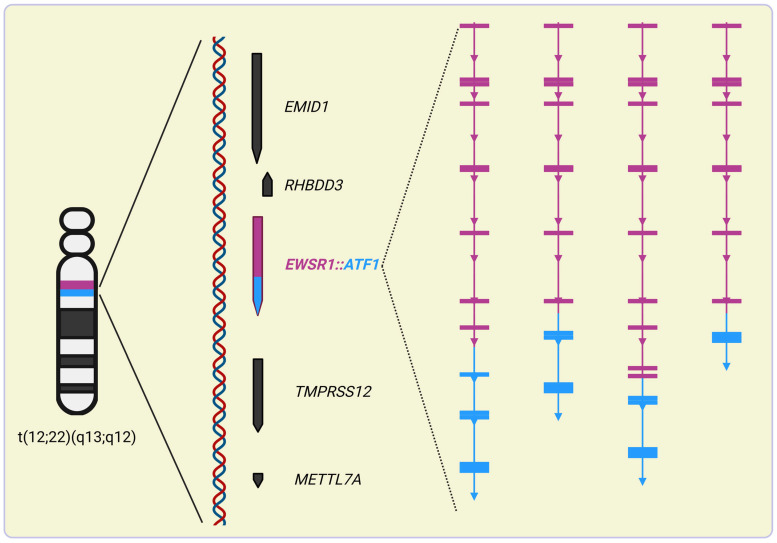
Variants of known *EWSR1::ATF1* fusion transcripts found in clear cell carcinomas. Numbers indicate exons of *EWSR1* and *ATF1*. Different colors of transcript variants represent spliced neighboring exons. The figure was created with Biorender.com.

**Figure 5:**
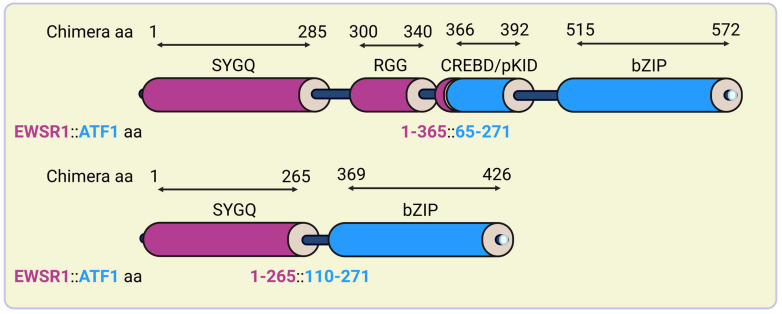
Structure of the most common EWSR1::ATF1 in CCS. EWSR1::ATF1 contains the N- and C-terminal regions of EWSR1 and ATF1, respectively. Black numbers represent amino acids of the full-length chimera, color-coded numbers refer to the portions of EWSR1 (red) and ATF1 (blue) fused in the chimeric proteins. The figure was created with Biorender.com.

In *EWSR1::ATF1* chromosomal rearrangements, intronic chromosomal breakpoints most often fall in the 13,448 bp long intron-3 of *ATF1*. One can speculate that this is likely due to repetitive elements, including 19 Alu repeats, one LINE-1, one L3/CR1 and two MER1-type elements, that consist of 83% of intron-3 [86]. In *EWSR1::ATF1*-positive CCS cells, the in-frame junction of exon-8 of *EWSR1* (corresponding to nucleotide 1016 or codon 365 of *EWSR1* cDNA) to exon-4 of *ATF1* (corresponding to nucleotide 385 or codon 65 of *ATF1* cDNA) was found (Figure 4) [112]. The literature data suggest that this rearrangement is the most common in-frame translocation upon the genesis of *EWSR1::ATF1* (Figure 5). Additional variants of in-frame rearrangements include the junction of *EWSR1* exon-7 (corresponding to nucleotide 836 or codon 265 of *EWSR1* cDNA) to exon-5 of *ATF-1* (corresponding to nucleotide 519 or codon 110 of *ATF1* cDNA) (Figure 5) [113,114]. The latter one has also been described as being fused in-frame to exon-10 of *EWSR1* [115]. In hyalinizing clear cell carcinoma, it was also found that exon-11 of *EWSR1* can be fused in-frame to exon-3 of *ATF1* [116]. In contrast, certain rearrangements result in out-of-frame fusions—like the fusion of exon-7 of *EWSR1* to exon-7 of *ATF1*—that frequently accompanied in-frame fusions [86].

In a recent study, it was found that a significant proportion of patients harboring *EWSR1::ATF1* fusions have reciprocal rearrangements, resulting in *ATF1::EWSR1* transcripts as well. Reciprocal transcripts, however, seem to be out-of-frame due to a deletion or splicing out of exon-9 of *EWSR1* or by the insertion of four nucleotides at the fusion positions [15,86]. Sequencing of *ATF1::EWSR1* cDNA fragments showed that *ATF1* exon-3 can be fused with *EWSR1* exon-10, resulting in out-of-frame chimeric transcripts [86]. Similarly, nucleotide 428 of *ATF1* exon-4 has been shown to be fused with *EWSR1* exon-8 and an insertion of four nucleotides (TGCA) at the junction renders the chimeric transcript out-of-frame [86]. Bioinformatics of the deduced translation products of the reciprocal transcripts predict a protein composed of the first 65 amino acids of ATF1 followed by 3 amino acids (threonine, histidine and glutamine) before the stop codon [86]. These data suggest that reciprocal *ATF1::EWSR1* transcripts are not involved in tumorigenesis.

As a result, in-frame *EWSR1::ATF1* produces the oncogenic EWSR1::ATF1 chimeric protein, which is the cytogenetic hallmark of certain soft-tissue tumors and is often found as the only chromosomal abnormality in the affected neoplasms [15,17,117]. In general, translated fusion proteins are believed to bind to ATF sites present in cAMP-responsive promoters via the bZIP domain of ATF1 and activate transcription constitutively [118]. However, data indicate that the genesis of EWSR1::ATF1 promotes ATF1 retargeting to novel consensus binding sites. Indeed, while about two-thirds of wild-type ATF1 binding sites are located at promoter regions, around 80% of the EWSR1::ATF1 binding sites occur distally [119]. While the fusion protein continues to operate through cAMP-responsive elements, the presence of the EWSR1 domain fosters greater transcription activity likely through the displacement of ATF1 and recruitment of additional transcription factors, such as SOX10, TFAP2A and MITF [119]. In addition, EWSR1::ATF1 interacts with additional motifs that, typically, are not recognized by wild-type ATF1, like the TGA repeats or AP1 motifs, that can further contribute to the dysregulation of gene expression [120].

Since the bZIP domain of wild-type ATF1 is phosphorylated by CKII, CKII-mediated enhancement of the DNA binding of ESWR1::ATF1 might be preserved in the functional chimeras too [94]. In contrast, the pKID of wild-type ATF1 is either excluded or truncated in EWSR1::ATF1 chimeras, suggesting that their oncogenic activity is not dependent on PKA-mediated signaling (Figure 5).

The absence of *EWSR1* exon-8 in certain fusion variants removes the EWSR1 IQ domain of which phosphorylation appears to enhance EWSR1::ATF1 activity [69]. Similarly, the absence of codons 65 to 109 of *ATF1* in some *EWSR1::ATF1* fusion products, apparently, excludes the putative activation domain of ATF1, raising the question of if the differential activity and, consequently, tumor specificity of these variants correlates to the tumor phenotype [121].

The comprehensive list of target tumor-driver genes of EWSR1::ATF1 is not known. The observation that EWSR1::ATF1-positive clear cell sarcomas show consistent melanocytic differentiation and expression of the melanocytic-specific *MITF* (*MITF-M*) transcript fueled the idea of the EWSR1::ATF1 fusion protein-mediated transactivation of the *MITF* promoter [112,122,123]. Consistently high expression of *MITF* and other key genes involved in melanogenesis, like the tyrosinase-related protein 1 (TYRP1), CDK2 and human homologue of murine silver (SILV/PMEL17/GP100), in EWSR1::ATF1 fusion-positive soft-tissue clear cell sarcomas further suggests that genes of melanin metabolism are bona fide targets of EWSR1::ATF1 chimeras, at least, in CCS. In AFH, however, the melanin metabolism-related genes are not expressed despite the presence of functional EWSR1::ATF1, indicating cell-type-specific target gene sets of EWSR1::ATF1 chimeras [124].

## 6. Clinical Presentation of the EWSR1::ATF1 Fusion

EWSR1::ATF1 fusion is linked to numerous malignancies spanning a wide age range. Although EWSR1::ATF1-positive cancers are usually rare pathologies, they include histopathologically different and particularly malignant neoplasms like the two pediatric central nervous system cancers, the intracranial non-myxoid angiomatoid fibrous histiocytoma (iAFH) and the atypical central neurocytoma [18,19]. iAFH is, typically, more frequent in females in their 20s, although cases have been reported at later ages as well [18]. Clinical presentation like headache and diplopia is nonspecific and refers to the intercranial hypertension only [18]. Lesions, which often contain increased amounts of lipids, choline and show large pseudo-vascular spaces without necrosis, have thick collagen fibers and epithelioid cells with nuclei containing open chromatin that show nonspecific immunohistochemistry positivity [18]. Due to these nonspecific characteristics, detection of EWSR1::ATF1 can be particularly useful for the histopathological diagnosis of iAFH.

Compared to AFH, the central neurocytoma is more aggressive due to focal necrosis and vascular proliferation that are usually present in this neoplasm [19]. Central neurocytoma typically affects the lateral ventricles in children [19]. Macroscopically, the tumor is soft with necrosis and large areas of hemorrhage [125]. Microscopically, it contains polygonal cells with round “salt and pepper” nuclei [126]. Mitotic figures are often present as well [126].

The typical clinical presentation of clear cell sarcoma (CCS) is a painful, rapidly growing mass around the lower extremities or neck of the patient [127]. CCS is a soft-tissue tumor that contains epithelioid cells with distinct cytoplasmic boundaries with granular nuclei [127,128]. Cells are in a nest formation with no clear nuclear pleomorphism or mitotic figures [127,128]. There is also no evidence of necrosis in the tumor [127]. It has a very poor prognosis; half of the patients develop distant malignancies and the 5- and 10-year survival rates are only 50% and 38%, respectively [16]. Traditional chemo- and radiotherapies have minimal benefit in CCS, although a pan histone deacetylase inhibitor, Vorinostat, has been found effective in repressing *EWSR1::ATF1* expression by an unknown mechanism [16]. CCS was originally thought to be melanoma due to its melanocytic differentiation characteristics, likely due to the expression of genes of melanin metabolism driven by the EWSR1::ATF1 chimera as discussed above. Molecular analyses revealed that, in almost all instances, CCS is positive for t(12;22)(q13;q12), a genetic event not seen in traditional malignant melanoma, that now provides a measure for differential molecular diagnosis [129].

The EWSR1::ATF1-driven expression of melanocytic markers such as HMB45, Melan A and tyrosinase can also serve in the differential diagnosis of gastrointestinal clear cell sarcomas from other aggressive *EWSR1::ATF1*-positive malignancies of the GI tract like gastrointestinal neuroectodermal tumors (GNETs).

GNET tumors stain positive for S-100 protein, CD56, NB84, synaptophysin, NSE and SOX10, while lacking melanocyte-specific immunomarkers. Microscopically, these tumors exhibit neural differentiation, such as synaptic-like vesicles [130]. Histologically, GNET tumor cells are arranged in sheets or nests, with a primitive oval- to spindle-shaped morphology, containing nuclei with vesicular chromatin and prominent nucleoli [130,131]. The cytoplasm appears pale eosinophilic or clear and osteoclast-type giant cells are observed in 50% of cases [130,131]. Clinical presentation is nonspecific with symptoms of abdominal pain, distention, weight loss, anemia or abdominal mass on imaging [131]. GNETs involve the small bowel, stomach or colon and, by the time of clinical symptoms, they are usually metastasized to regional lymph nodes or the liver, resulting in a median survival time of approximately 18 months [132,133].

Pediatric malignant mesothelioma, which is usually peritoneal and pericardial, is also known to harbor *EWSR1::ATF1* rearrangements [17]. Since these rearrangements are not frequently seen in malignant mesothelioma with adult onset, which is believed to be tightly correlated with asbestos or radiation exposure, the *EWSR1::ATF1*-positive pediatric cases are believed to present a distinct subtype of malignant mesotheliomas [17].

EWSR1::ATF1 fusions are frequently observed in malignant testicular sex cord tumors as well that display distinct morphologic and immunophenotypic features from Sertoli cell tumors [134]. A histologic section of a nested testicular sex cord tumor primarily reveals a solid architecture, with some regions arranged in nests or net-like patterns and variable fibrotic stroma. Focal areas of necrosis and a varying aggregate of inflammatory cells, predominantly lymphocytes and plasma cells, are often observed [134]. Tumor cells display eosinophilic, lipidized cytoplasm and relatively monomorphic nuclei, with scattered larger nuclei exhibiting atypia and increased mitotic figures [20]. Immunohistochemistry stains positive for SF-1, EMA, CD30, WT1 and inhibin. Although testicular sex cord tumors generally confer good prognosis, metastases significantly worsen outcomes due to their poor response to systemic treatments [134].

## 7. Discussion

The concept that chromosomal damage could serve as a predictor of neoplasm development stems from the perception that genetic damage observed in peripheral lymphocytes reflects similar events occurring in precursor cells undergoing tumorigenesis irrespective of carcinogen exposures [135,136].

Specific chromosomal changes predict different neoplasm hallmarks, including increased cellular proliferation and immune evasion, associated with focal and whole-chromosome aneuploidy, respectively [137]. This distinction arises from the degree of gene involvement. Focal aneuploidies affect specific chromosomal segments that directly influence the cell cycle, which strongly promotes proliferation. In contrast, whole-chromosome aneuploidies impact a larger number of genes, many of which may not directly influence proliferation. However, these broader changes can induce proteotoxic stress due to gene dosage imbalance, ultimately hindering cell proliferation [137]. A negative correlation exists between aneuploidy and immune response across many cancer types, potentially due to factors such as proteotoxic stress, weakened antigen presentation on the major histocompatibility complex and reduction in neoantigen concentrations. Indeed, assessing aneuploidy along with mutation loads provides valuable predictions for immunotherapy response and overall patient survival [137].

Chromosome segregation errors not only result in numerical aneuploidy but also precipitate unbalanced translocations due to cytokinesis-induced DNA double-strand breaks (DSB), arm-level segmental imbalances or the formation of micronuclei [138]. Cytokinesis force can generate chromosome fragments that are distributed to distinct daughter cells, potentially leading to the subsequent abnormal rearrangement of genetic material between chromosomes [139]. Micronuclei are encapsulated abnormal structures comprised of dysfunctional nuclear envelopes that facilitate DSB accumulation during the interphase. Faulty compactization throughout the interphase allows for extensive chromosome fragmentation in the subsequent mitosis. Rearrangements of fragmented chromosomes can generate alterations and mutations that enable oncogenic selective advantages for the cancer genome [138].

Despite its small size, chromosome 22 is associated with a number of human pathologies including the CML, Burkitt’s lymphoma, meningiomas and malignancies of the nervous system. The *EWSR1* of chromosome 22 provides the prototype of fusion oncogenes including the form of *EWSR1::ATF1* [140]. It acts as an aberrant transcription factor in which EWSR1 provides the transcriptional activation motif while ATF1 contributes to the fusion protein with its own DNA-binding domain. This domain configuration fueled the idea that EWSR1::ATF1 chimeras act like aberrant transcription factors, which dysregulate the gene expression pattern of host cells, leading to unleashed proliferation and, thus, tumor formation. Transfection experiments with other, structurally related EWSR1 chimeras, like *EWSR1::FLI1*, support this concept, although the detailed underlying mechanism, including the list of chimera-dysregulated tumor-driver genes, is still to be elucidated.

Indeed, molecular analyses of genes fused in these chimeras raise, probably, more questions than answers. For instance, it is currently unknown if transcriptional reprogramming by the chimeric proteins or the disrupted wild-type EWSR1 functions in RNA metabolism and/or DNA repair is more important to carcinogenesis.

Also, we know that EWSR1 chimeras retain most, but not necessarily all, of the low complexity domain of EWSR1, raising the question of if LCD-mediated phase separation is disturbed upon the appearance of fusion proteins. Indeed, phase separation can fundamentally determine the localization of proteins, and it is believed that FET family members have functions in RNA processing that require their nuclear localization. Comparison of FET family members FUS and EWSR1 revealed that they share very few gene targets, suggesting that they are not redundant [39]. Thus, one can speculate that the loss of critical wild-type EWSR1 activities in the regulation of gene expression, like the repression of *PRAS40* and *CFL-1* mRNAs that was shown to inhibit proliferation, migration and invasion of Ewing sarcoma family tumors, cannot be fully compensated by other FET members [55,56]. Similarly, dislocation of EWSR1 chimeras could easily affect the role of EWSR1 in the regulation of RNS metabolism by its own.

Phosphorylation of FUS by kinases such as ATM and CDK1/2 can influence its RNA- and DNA-binding capacity, thereby impacting its putative roles in RNA splicing and DNA damage response [141]. Phosphorylation of the N-terminus of FUS specifically initiates its translocation to the cytoplasm so one can speculate if any disturbance in DNA repair-related post-translational modification of related proteins, including EWSR1, due to their intracellular misplacement in their chimeric forms, might turn them into mutator genes [141].

Mapping the chromosomal environments of loci involved in t(12;22)(q13;q12) rearrangements reveals that *EWSR1* and *ATF1* are flanked by *RHBDD3*, and *TMPRSS12*, respectively [72]. Interestingly, both *RHBDD3* and *TMPRSS12* are predicted to encode a serine-type endopeptidase activity, so one can speculate if t(12;22)(q24;q12) might affect intracellular serine-type endopeptidase activity by disturbing the trans-regulatory elements-mediated transcriptional regulation of *RHBDD3* and/or *TMPRSS12*.

The frequent presence of *EWSR1* locus abnormalities in a wide range of histopathologically distinct human neoplasms mandate *EWSR1* chimeras as valid therapeutic targets for tumor-agnostic therapeutic measures. Indeed, the first steps have already been taken for its use in the laboratory diagnostics context. In hepatocellular carcinoma (HCC), EWSR1 was identified as upregulated [142]. Since some of the current HCC immunohistochemistry biomarkers, like CCL14 and CK19, frequently result in inaccurate staining, EWSR1 has been proposed to be used as a nuclear staining biomarker for HCC for more conclusive results [142]. For effective tumor-agnostic therapeutic agents designed against EWSR1-related biomarkers, however, more detailed understanding of EWSR1 rearrangements, including the t(12;22)(q13;q12) translocation, is needed. These efforts, hopefully, will not only elucidate the above fascinating scientific dilemmas but also shed more light on the fine details of the enormous complexity of the regulation of human gene expression.
